# Clinical Outcomes of Conversion Surgery after Neoadjuvant Chemotherapy in Patients with Borderline Resectable and Locally Advanced Unresectable Pancreatic Cancer: A Single-Center, Retrospective Analysis

**DOI:** 10.3390/cancers11030278

**Published:** 2019-02-26

**Authors:** Changhoon Yoo, Sang Hyun Shin, Kyu-pyo Kim, Jae Ho Jeong, Heung-Moon Chang, Jun Ho Kang, Sang Soo Lee, Do Hyun Park, Tae Jun Song, Dong Wan Seo, Sung Koo Lee, Myung-Hwan Kim, Jin-hong Park, Dae Wook Hwang, Ki Byung Song, Jae Hoon Lee, Baek-Yeol Ryoo, Song Cheol Kim

**Affiliations:** 1 Department of Oncology, Asan Medical Center, University of Ulsan College of Medicine, Seoul 05505, Korea; cyoo.amc@gmail.com (C.Y.); kkp1122@gmail.com (K.-p.K.); imdrho@gmail.com (J.H.J.); changhm@amc.seoul.kr (H.-M.C.); bodle1125@naver.com (J.H.K.); 2 Department of Surgery, Asan Medical Center, University of Ulsan College of Medicine, Seoul 05505, Korea; surgeonssh@gmail.com (S.H.S.); dwhwang@amc.seoul.kr (D.W.H.); mtsong21c@naver.com (K.B.S.); hbpsurgeon@gmail.com (J.H.L.); 3 Department of Surgery, Samsung Medical Center, Sungkyunkwan University School of Medicine, Seoul 06351, Korea; 4 Department of Gastroenterology, Asan Medical Center, University of Ulsan College of Medicine, Seoul 05505, Korea; ssleedr@amc.seoul.kr (S.S.L.); dhparkeus@gmail.com (D.H.P.); medi01@naver.com (T.J.S.); dwseoamc@amc.seoul.kr (D.W.S.); sklee@amc.seoul.kr (S.K.L.); mhkim@amc.seoul.kr (M.-H.K.); 5 Department of Radiation Oncology, Asan Medical Center, University of Ulsan College of Medicine, Seoul 05505, Korea; pjhynwie@hanmail.net

**Keywords:** pancreatic cancer, neoadjuvant chemotherapy, borderline resectable pancreatic cancer, locally advanced pancreatic cancer

## Abstract

The clinical benefit and potential risks of conversion surgery after neoadjuvant chemotherapy (NACT) have not been fully investigated in patients with borderline resectable pancreatic cancer (BRPC) and locally advanced unresectable pancreatic cancer (LAPC). Therefore, this has been evaluated in a retrospective, prospective cohort-based analysis. Between October 2005 and April 2017, 135 patients (65 with BRPC and 70 with LAPC) received conversion surgery after NACT. Exploratory analysis to assess clinical outcomes in comparison with patients who underwent upfront surgery in the same time period (*n* = 359) was also conducted. NACT with gemcitabine-based regimens (including gemcitabine monotherapy, gemcitabine-capecitabine combination, and gemcitabine-erlotinib combination) was used in 69 patients (51%) and FOLFIRINOX in 66 patients (49%). The median overall survival (OS) and disease-free survival (DFS) from the time of surgery was 25.4 months (95% CI, 18.6–32.2 months) and 9.0 months (95% CI, 6.8–11.2 months), respectively. The median OS and progression-free survival from the initiation of NACT was 29.7 months (95% CI, 22.5–36.8 months) and 13.4 months (95% CI, 12.5–14.4 months), respectively. In the exploratory analysis, conversion surgery after NACT was associated with a better median OS and DFS than upfront surgery (vs. 17.1 months; 95% CI, 15.5–18.7 months; *p* = 0.001 and vs. 7.1 months; 95% CI, 6.4–7.8 months; *p* = 0.005, respectively). There was no difference in length of hospital stay between the two groups, and conversion surgery after NACT showed a significantly lower incidence of postoperative complications than upfront surgery (38% vs. 27%, *p* = 0.03). Conversion surgery after NACT is a feasible and effective therapeutic strategy for the treatment of patients with BRPC and LAPC. Further clinical trials investigating optimal therapeutic strategies for BRPC and LAPC are warranted.

## 1. Introduction

The poor prognosis of pancreatic cancer, with a 5 year survival rate of less than 6%, is well-known [1,2]. Although surgical resection is the only potentially curative therapy, only 10–20% of patients with pancreatic cancer are classified as having resectable disease at the time of diagnosis [3]. Locally advanced, non-metastatic pancreatic cancer occurs in approximately 30% of newly diagnosed patients [3] and includes borderline resectable pancreatic cancer (BRPC) and locally advanced unresectable pancreatic cancer (LAPC) [4]. Multiple criteria have been proposed to define BRPC and LAPC by groups such as the National Comprehensive Cancer Network (NCCN), the joint consensus conference of the Americas Hepato-Pancreato-Biliary Association (AHPBA), the Society of Surgical Oncology (SSO), and the Society for Surgery of the Alimentary Tract (SSAT) [5,6].

Although neoadjuvant chemotherapy (NACT) has previously been investigated for the management of BRPC and LAPC with the expectation that it may lead to downstaging, no practice-changing data were ever generated, mainly because of low response rates to conventional chemotherapeutic regimens [7,8]. However, in recent years, the emergence of more effective chemotherapy regimens, such as FOLFIRINOX (fluorouracil, leucovorin, irinotecan, and oxaliplatin) and gemcitabine plus *nab*-paclitaxel, has renewed interest in the use of NACT in patients with BRPC and LAPC [9,10].

Therefore, we conducted a retrospective analysis to evaluate the clinical outcomes of conversion surgery after NACT in patients with BRPC and LAPC. We also performed an exploratory analysis to measure the survival benefit and potential risks associated with conversion surgery after NACT, in comparison with upfront surgery.

## 2. Patients and Methods

### 2.1. Patients

A total of 1888 patients with pancreatic ductal adenocarcinoma (PDAC) who underwent surgical resection between October 2005 and April 2017 were identified in the prospective database of the Department of Surgery, Asan Medical Center, Seoul, Korea. Among them, 179 patients underwent surgical resection after chemotherapy. After the exclusion of patients who had distant metastasis (M1) or resectable disease at baseline, 135 patients (65 with BRPC and 70 with LAPC) were identified who had received conversion surgery after NACT and were included in this study, as shown in Supplementary Figure S1. For exploratory analysis to compare the clinical outcomes between conversion surgery after NACT and upfront surgery, a patient cohort who had received upfront surgery was also identified. In the same period, 1709 patients underwent upfront surgery, among whom 359 had received surgery for BRPC (*n* = 281) or LAPC (*n* = 78) and were included in the exploratory analysis. The study was approved by the institutional review board of the Asan Medical Center, Seoul, Korea (IRB approval number: 2016-0902).

Baseline characteristics, pathology, and clinical outcomes were acquired from the prospective database. Baseline imaging studies, including computed tomography (CT), magnetic resonance imaging (MRI), and FDG-positron emission tomography (PET)-CT, were reviewed retrospectively. In this study, BRPC and LAPC were classified according to the NCCN resectability criteria [5].

### 2.2. Endpoints and Statistical Analysis

Responses were graded according to the Response Evaluation Criteria for Solid Tumors (RECIST) version 1.1 [11]. Surgical complications and postoperative pancreatic fistula were graded using the Clavien–Dindo classification system [12] and guidelines of the International Study Group on Pancreatic Fistula (ISGPF) [13], respectively. Disease-free survival (DFS) was defined as the time from surgery to the date of recurrence or death, whichever occurred first. Overall survival (OS) was estimated as the time between surgery and any cause of death. In the conversion surgery after NACT group, progression-free survival (PFS) was defined as the time from NACT to the date of recurrence or death, whichever occurred first.

Categorical variables were compared using the chi-square or Fisher’s exact test, as appropriate. Survival curves were estimated by the Kaplan–Meier method and compared by log-rank tests. Cox-proportional hazards models were used for univariate and multivariate analyses, and outcomes are shown with the hazard ratio (HR) and 95% confidence interval (CI). The variables with potential significance (*p* < 0.2) in the univariate analyses were included in the multivariate analysis, using a backward likelihood method to define the prognostic factors of conversion surgery after NACT. To adjust for potential confounding variables in the impact of NACT, multivariate analysis using the Enter method was conducted with the inclusion of NACT and key prognostic factors. Because T and N stages, resection margin status, and vascular resection status are affected by NACT, these variables were not included considering the multicollinearity. All statistical analyses were performed using the Statistical Package for the Social Sciences (IBM SPSS, Chicago, IL, USA) version 21.0, and all tests were two-sided with 5% defined as the level of significance.

## 3. Results

### 3.1. Patient Characteristics

Baseline patient characteristics are summarized in Table 1; the median age was 60 years (range, 30–78 years); 53% were male in the conversion surgery after NACT group. According to the NCCN criteria, 65 patients (48%) were classified as BRPC and 70 (52%) as LAPC at the time of diagnosis. As NACT, 69 patients (51%) received gemcitabine-based regimens, including gemcitabine monotherapy, gemcitabine-capecitabine combination, and gemcitabine-erlotinib combination; 66 patients (49%) received FOLFIRINOX. According to RECIST v1.1, the best responses to NACT were partial response, stable disease, and progressive disease in 52 (39%), 80 (59%), and 3 (2%) patients, respectively. The median duration of NACT was 3.0 months (range, 0.1–11.6 months). Postoperative chemotherapy and radiotherapy were administered in 105 (78%) and 18 (13%) patients, respectively.

No significant differences in gender or age were seen between the conversion surgery after NACT and upfront surgery groups, as shown in Table 1. The patients in the conversion surgery after NACT group had more advanced disease at the time of diagnosis than did those in the upfront surgery group, as there was a significant difference in the proportion of BRPC and LAPC between the two groups (BRPC/LAPC: 48/52% in the conversion surgery after NACT group vs. 78/22% in the upfront surgery group; *p* < 0.001). More patients in the upfront surgery than in the conversion surgery after NACT group underwent extensive surgery, including total pancreatectomy (21% vs. 11%) or major venous resection (89% vs. 59%), although the rate of major arterial resection was lower in the upfront surgery group (20% vs. 29%). At the time of surgical resection, CA 19-9 levels were elevated more frequently in the upfront surgery group than in the conversion surgery after NACT group (72% vs. 56%, *p* = 0.001). There was no difference between the two groups in length of hospital stay for surgery (upfront surgery vs. conversion surgery after NACT, 17 vs. 13 days, *p* = 0.14), postoperative chemotherapy (69% vs. 78%, *p* = 0.06), or postoperative radiotherapy (21% vs. 13%, *p* = 0.05). The details of postoperative chemotherapy were available in 228 patients in the upfront surgery group and 88 patients in the conversion surgery after NACT group, except those who received postoperative chemotherapy in outside hospitals. Fluoropyrimidine (*n* = 168, 74%), gemcitabine (*n* = 56, 24%), gemcitabine-capecitabine combination (*n* = 2, 1%), and gemcitabine-*nab*-paclitaxel combination (*n* = 2, 1%) were used as postoperative chemotherapy in the upfront surgery group, and gemcitabine (*n* = 42, 48%), fluoropyrimidine (*n* = 20, 23%), gemcitabine-capecitabine combination (*n* = 12, 13%), FOLFIRINOX (*n* = 13, 15%), and gemcitabine-*nab*-paclitaxel combination (*n* = 1, 1%) in the conversion surgery after NACT group. The median duration of postoperative chemotherapy was 3.2 months (range, 0.5–9.0 months) and 3.3 months (range, 0.5–9.5 months) in the upfront surgery and conversion surgery after NACT groups, respectively.

### 3.2. Postoperative Stage and Morbidity

Postoperative pathological staging is summarized in Table 2. Compared with the upfront surgery group, the conversion surgery after NACT group showed less advanced T stage (T3–4, 93% vs. 99%, *p* = 0.001), N stage (N+, 49% vs. 71%, *p* < 0.001), lymphovascular invasion (36% vs. 58%, *p* < 0.001), and perineural invasion (80% vs. 94%, *p* < 0.001) as shown in Table 2. The R1 resection rate was higher in the upfront surgery group than in the conversion surgery after NACT group (33% vs. 24%), and this difference was marginally significant (*p* = 0.06). There was no patient with R2 resection in both groups.

In the conversion surgery after NACT group, surgical complications were noted in 37 patients (27%), and graded as I–II, III–IV, and V in 24 (18%), 12 (9%), and 1 (1%) patient, respectively, as shown in Table 3. Clinically relevant postoperative pancreatic fistula (grade B or C) occurred in 14 patients (10%). Overall postoperative morbidity (Clavien–Dindo grade I–V) was more frequent in the upfront surgery group than in the conversion surgery after NACT group (38% vs. 27%, *p* = 0.03), while clinically relevant postoperative pancreatic fistulae (grade B or C) were more frequent in the conversion surgery after NACT group than in the upfront surgery group (10.4% vs. 4.2%, *p* = 0.02).

### 3.3. Survival Outcomes and Prognostic Factors with Conversion Surgery after NACT

**Figure 1:**
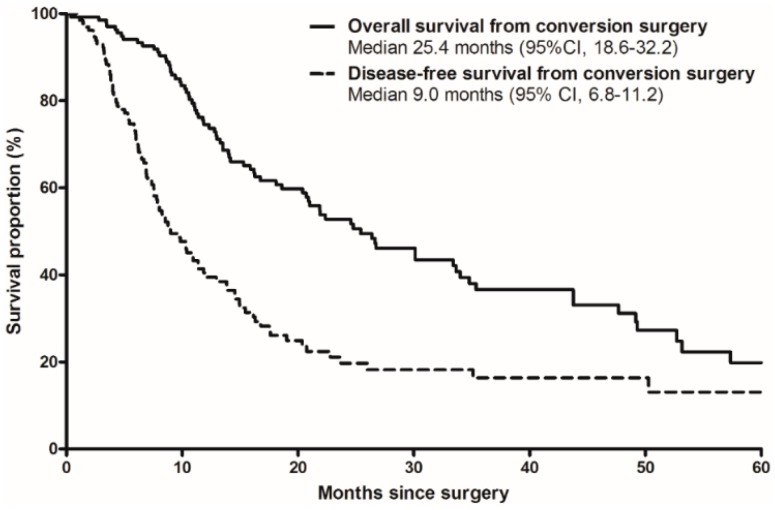
Overall survival (solid line) and disease-free survival (dotted line) from the time of surgery in patients with conversion surgery after neoadjuvant chemotherapy.

The median OS from the time of surgery was 25.4 months (95% CI, 18.6–32.2 months), and 2 and 4 year OS rates were 52.8% (95% CI, 43.8–61.8%) and 31.2% (95% CI, 21.2–41.2%), respectively, as shown in Figure 1. The median DFS from the time of surgery was 9.0 months (95% CI, 6.8–11.2 months), and 2 and 4 year DFS rates were 18.2% (95% CI, 10.4–26.0%) and 13.1% (95% CI, 4.7–21.5%), respectively.

Univariate and multivariate analyses of OS and DFS from surgery in the conversion surgery after NACT group were performed with the inclusion of age, gender, pT stage, pN stage, surgical type, NACT regimens, objective response by RECIST v1.1, CA 19-9 levels, major vascular resection, and resection margin status, as shown in Table 4. NACT regimens (gemcitabine-based chemotherapy vs. FOLFIRINOX) showed no statistically significant differences in terms of OS or DFS from surgery (*p* = 0.31 and *p* = 0.76, respectively). However, a statistically significant association was seen between age (>65 vs. <65 years; HR, 2.7 (95% CI, 1.4–5.2); *p* = 0.003) and response to NACT (progressive disease vs. partial response; HR, 2.1 (95% CI, 1.2–3.9); *p* = 0.02; stable disease vs. partial disease; HR, 4.9 (95% CI, 1.1–23.0); *p* = 0.04) and OS. A statistically significant association was seen between DFS and CA 19-9 level at surgery (elevated vs. normal; HR, 1.6 (95% CI, 1.00–2.7); *p* = 0.049) and resection of major veins (with resection vs. without resection; HR, 1.7 (95% CI, 1.00–2.8); *p* = 0.047).

**Figure 2:**
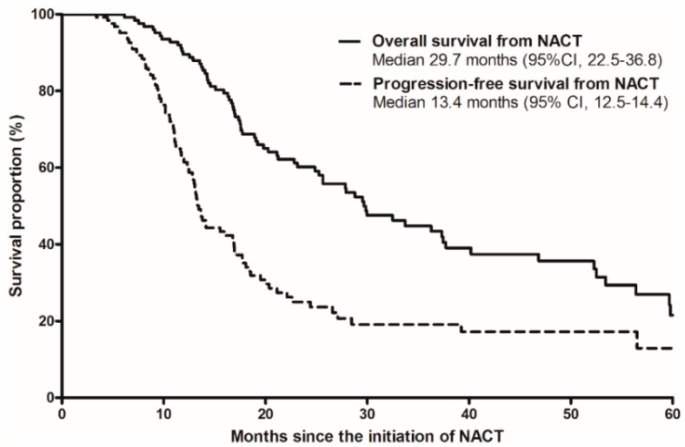
Overall survival (solid line) and progression-free survival (dotted line) from the initiation of neoadjuvant chemotherapy surgery in patients with conversion surgery after neoadjuvant chemotherapy (NACT).

The median OS and PFS from the initiation of NACT was 29.7 months (95% CI, 22.5–36.8 months) and 13.4 months (95% CI, 12.5–14.4 months), respectively, as shown in Figure 2. The 2 and 4 year OS rates from the initiation of NACT were 60.2% (95% CI, 51.2–69.2%) and 35.6% (95% CI, 25.4–45.8%), respectively, and the 2 and 4 year PFS rates from the initiation of NACT were 25.0% (95% CI, 16.4–33.6%) and 17.2% (95% CI, 9.0–26.2%), respectively.

### 3.4. Survival Outcomes with Conversion Surgery after NACT in Comparison with Upfront Surgery

**Figure 3:**
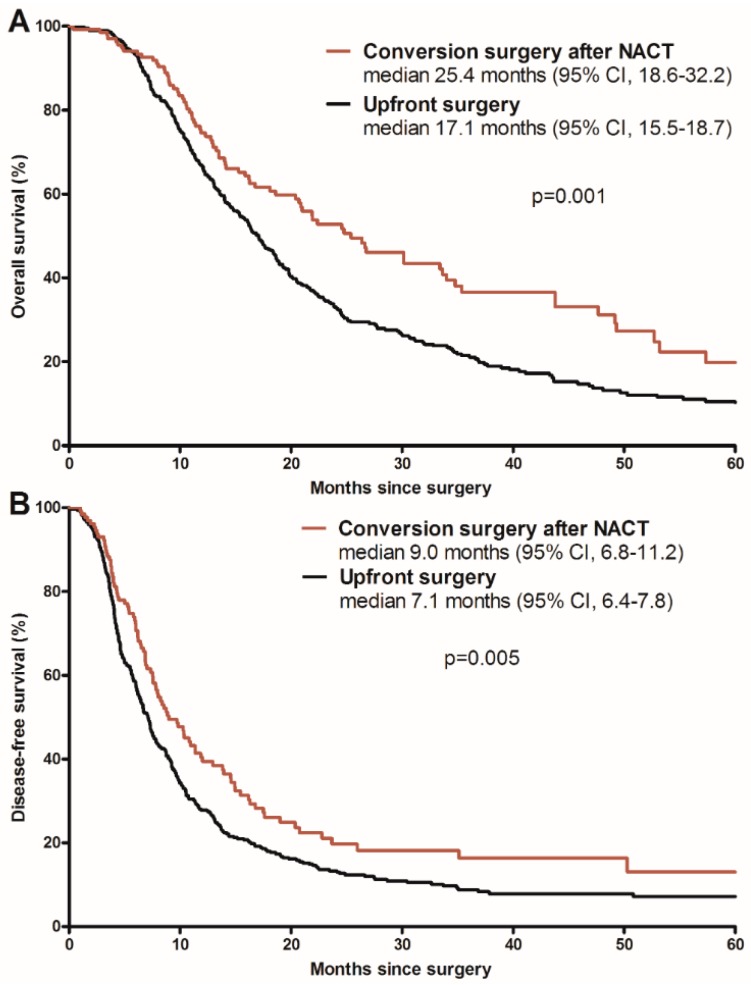
Comparison of overall survival (**A**) and disease-free survival (**B**) between patients undergoing conversion surgery after neoadjuvant chemotherapy (NACT) or upfront surgery.

The median follow-up duration from surgery was 17.1 months (range, 0.5–129.9) in the upfront surgery group and 17.5 months (range, 0.4–128.9) in the conversion surgery after NACT group, with no statistically significant differences seen between the groups (*p* = 0.58). Compared with upfront surgery, the conversion surgery after NACT group showed better OS from surgery (25.4 months vs. median 17.1 months (95% CI, 15.5–18.7 months); unadjusted HR, 0.67 (95% CI, 0.52–0.85); *p* = 0.001), as shown in Figure 3A, and DFS from surgery (9.0 months vs. 7.1 months (95% CI, 6.4–7.8 months); unadjusted HR, 0.72 (95% CI, 0.57–0.91); *p* = 0.005), as shown in Figure 3B. In multivariate analyses for OS and DFS, conversion surgery after NACT remained significantly superior to upfront surgery in terms of OS and DFS (HR, 0.73 (0.56–0.96); *p* = 0.02; and HR, 0.72 (0.56–0.93); *p* = 0.01, respectively), as shown in Table 5.

In the subgroup analyses for the BRPC cohort, median OS and DFS in the conversion surgery after NACT group were 21.9 months (95% CI, 15.8–27.9 months) and 8.8 months (95% CI, 6.0–11.6 months), respectively, while they were 16.9 months (95% CI, 14.7–19.1 months; *p* = 0.03) and 7.3 months (95% CI, 6.4–8.2 months; *p* = 0.08) in the upfront surgery group, respectively. For the LAPC cohort, median OS and DFS in the conversion surgery after NACT group were 26.6 months (95% CI, 19.3–34.0 months) and 9.8 months (95% CI, 5.5–14.2 months), respectively, and in the upfront surgery group, 17.1 months (95% CI, 14.3–19.9 months; *p* = 0.01) and 6.1 months (95% CI, 4.8–7.4 months; *p* = 0.01), respectively.

## 4. Discussion

The present study showed that conversion surgery after NACT is feasible and effective in patients with BRPC and LAPC at the time of diagnosis. The median OS and DFS from conversion surgery in the study cohort were 25.4 and 9.0 months, respectively. When the survival outcomes were estimated from the time of the start of NACT, the median OS and PFS were 29.7 and 13.4 months, respectively. These findings are in line with the results of a previous retrospective analysis [14] and suggest that patients who undergo conversion surgery after NACT for BRPC and LAPC may have similar survival outcomes to those with resectable pancreatic cancer [15].

Prognostic factors for patients undergoing conversion surgery after NACT were age, CA 19-9 levels, and response to NACT for OS, and CA 19-9 and resection of major veins (such as the portal vein and superior mesenteric vein) for DFS. Remarkably, patients who showed progressive disease on NACT showed poorer OS after conversion surgery. This may suggest that patients with progressive disease on NACT should be cautiously discussed in the multidisciplinary team for surgical resection even though R0 resection might be achieved considering the poor prognosis and potential negative impact on quality of life of the surgery. Interestingly, there was no difference in survival outcomes between gemcitabine-based regimens and FOLFIRINOX. Since gemcitabine plus *nab*-paclitaxel is not approved for the treatment of patients with BRPC or LAPC in Korea, all patients in this study received other gemcitabine-based regimens, including gemcitabine monotherapy, gemcitabine plus erlotinib, or gemcitabine plus capecitabine. Since this study included only patients who could undergo resection after some degree of response to NACT, it is not appropriate to compare the efficacy of different chemotherapy regimens as NACT in BRPC and LAPC in this patient population. Although previous studies showed favorable outcomes with FOLFIRINOX compared with conventional gemcitabine-based regimens [16,17,18,19], the optimal NACT regimen in patients with BRPC and LAPC should be investigated in prospective clinical trials. Ongoing randomized trials, including the ALLIANCE A021501 study, may help to define standard regimens for BRPC and LAPC [20].

The risk of postoperative complications is a potential issue for conversion surgery after NACT in patients with pancreatic cancer. Our findings indicate that conversion surgery after NACT is safe and may not increase postoperative complications in patients with BRPC and LAPC. There was also no difference in the length of hospital stay between conversion surgery after NACT and upfront surgery in our exploratory analysis cohort. Furthermore, patients with conversion surgery after NACT showed less postoperative morbidity than those with upfront surgery (24% vs. 33%), even though these patients had more extensive vascular involvement at the time of diagnosis. This finding is supported by a previous study [21] and might be attributable to the reduced extent of resection afforded by downstaging after NACT, since the major vascular resection rate was significantly lower in the conversion surgery after NACT group than in the upfront surgery group (73% vs. 98%, respectively). Although there is limited supporting preclinical evidence, these findings may be related to the fact that NACT may allow time for patients to recover from acute pancreatitis caused by cancer or biliary drainage procedures, or the potential impact of NACT on the peri-pancreatic cancer tissue, which allows for better suturing of the pancreaticojejunostomy.

In our exploratory analysis, we compared outcomes between conversion surgery after NACT and upfront surgery for BRPC and LAPC. Patients who received NACT before surgery showed less advanced T and N stages, and less frequent lymphovascular invasion and perineural invasion, than those with upfront surgery, despite the fact that patients who received NACT followed by surgery had a greater tumor burden at the time of diagnosis (proportion of LAPC, 52% vs. 22%). These results are in line with recent data for resectable pancreatic cancer [22]. Conversion surgery after NACT was significantly associated with better survival outcomes than upfront surgery in patients with BRPC and LAPC (OS, 25.4 vs. 17.1 months; DFS, 9.0 vs. 7.1 months). The differences in OS and DFS between the two groups remained significant in multivariate analyses, with similar adjusted HRs (0.73 for OS and 0.72 for DFS). Our finding is consistent with previous data from a large US national database of patients with resectable pancreatic cancer [22]. In that study, surgery after neoadjuvant treatment was associated with better OS than upfront surgery with an adjusted HR of 0.74.

Despite strong rationales for NACT, such as earlier eradication of micrometastasis and selection of patients with favorable tumor biology, and the promising results of NACT in BRPC and LAPC, controversies remain regarding the management of medically fit patients with technically resectable BRPC and LAPC. Since most data evaluating NACT followed by surgery, including those presented here, have analyzed highly selected populations that could tolerate and show response to NACT, there remains a lack of clear evidence to support the use of NACT in technically resectable BRPC and LAPC, particularly in cases where microscopic complete resection (R0) is highly achievable. With improvements in surgical technique and postoperative rehabilitation, more patients are able to receive adjuvant chemotherapy than before, and the efficacy of this approach has also recently shown remarkable improvement [15,23]. The advantages and disadvantages of NACT followed by surgery and upfront surgery followed by adjuvant chemotherapy for technically resectable BRPC and LAPC or resectable pancreatic cancer should be addressed in future randomized trials.

The present study has several limitations, including a retrospective design, a highly selected population obtained from a single tertiary institution, and an inability to address the clinical outcomes of patients with BRPC and LAPC who started chemotherapy but could not undergo surgical resection, which precluded a comprehensive assessment of the role for NACT in BRPC and LAPC. Despite these limitations, the strengths of this study are that our analyses were based on a large cohort and a prospectively established registry, which may reduce potential selection bias.

## 5. Conclusions

In conclusion, conversion surgery after NACT is a feasible and effective therapeutic strategy for patients with BRPC and LAPC. With recent advances in systemic chemotherapy and radiation therapy, future clinical trials investigating optimal therapeutic strategies for LAPC are strongly warranted.

## Figures and Tables

**Table 1:** Patient characteristics.

Variable	Upfront Surgery (*n* = 359)	Conversion Surgery after NACT (*n* = 135)	*p* Value
**Age, median (range)**	61 (30–82 years)	60 (30–78 years)	0.18
**Age ≥65 years**	132 (37%)	33 (24%)	0.01
**Gender, male/female**	202 (56%)/157 (44%)	72 (53%)/63 (47%)	0.18
**Resectability criteria at diagnosis**			
Borderline resectable	281 (78%)	65 (48%)	<0.001
Locally advanced unresectable	78 (22%)	70 (52%)	
**NACT regimens**			
Gemcitabine-based		69 (51%)	
FOLFIRINOX		66 (49%)	
**Response to chemotherapy**			
Partial response		52 (39%)	
Stable disease		80 (59%)	
Progressive disease		3 (2%)	
**Surgical type**			
Pancreatoduodenectomy	237 (66%)	80 (59%)	<0.001
Distal pancreatectomy	45 (13%)	40 (30%)	
Total pancreatectomy	77 (21%)	15 (11%)	
**Major vascular resection**	351 (98%)	99 (73%)	<0.001
Vein resection	324 (90%)	79 (59%)	
Artery resection	64 (18%)	39 (29%)	
Combined resection	37 (10%)	21 (16%)	
**CA 19-9 level at the time of surgery**	*N* = 351	*N* = 132	0.001
Within normal range	97 (28%)	58 (44%)	
Elevated	254 (72%)	74 (56%)	
**Hospital stay for surgery, median (interquartile range)**	17 days (11–24)	13 days (10–17)	0.14
**Postoperative adjuvant chemotherapy**	248 (69%)	105 (78%)	0.06
**Postoperative adjuvant radiotherapy**	76 (21%)	18 (13%)	0.05

NACT = neoadjuvant chemotherapy.

**Table 2:** Pathological features.

Variables	Upfront Surgery (*n* = 359)	Conversion Surgery after NACT (*n* = 135)	*p* Value
Pathological staging *			
T1–2/T3–4	5 (1%)/354 (99%)	10 (7%)/125 (93%)	0.001
T1	2 (0.6%)	8 (5.9%)	
T2	3 (0.8%)	2 (1.5%)	
T3	337 (93.9%)	119 (88.1%)	
T4	17 (4.7%)	6 (4.4%)	
N0/N1	104 (29%)/253 (71%)	69 (51%)/66 (49%)	<0.001
Resection margin status **			0.06
R0	240 (67%)	102 (76%)	
R1	119 (33%)	33 (24%)	
**Lymphovascular invasion**	209 (58%)	48 (36%)	<0.001
**Perineural invasion**	338 (94%)	108 (80%)	<0.001

NACT = neoadjuvant chemotherapy; * graded according to the American Joint Committee on Cancer (AJCC) staging 7th edition; ** there were no patients with R2 resection.

**Table 3:** Postoperative complications.

Variables	Upfront Surgery (*n* = 359)	Conversion Surgery after NACT (*n* = 135)	*p* Value
Surgical complication *	136 (38%)	37 (27%)	0.03
Grade I–II	110 (31%)	24 (18%)	
Grade III–IV	23 (6%)	12 (9%)	
Grade V	3 (1%)	1 (1%)	
Postoperative pancreatic fistula **	42 (12%)	20 (15%)	0.02
Biochemical leakage	27 (8%)	6 (4%)	
Grade B or C	15 (4%)	14 (10%)	

NACT = neoadjuvant chemotherapy; * graded using the Clavien–Dindo classification; ** graded using the guideline of the International Study Group on Pancreatic Fistula (ISGPF).

**Table 4:** Univariate and multivariate analyses of overall survival and disease-free survival in patients with conversion surgery after neoadjuvant chemotherapy.

Variables	Overall Survival				Disease-Free Survival			
	Univariate		Multivariate		Univariate		Multivariate	
	HR (95% CI)	*p*	HR (95% CI)	*p*	HR (95% CI)	*p*	HR (95% CI)	*p*
**Age (≥65 vs. <65 years)**	2.52 (1.24–5.13)	0.01	2.70 (1.39–5.23)	0.003	1.67 (0.69–3.13)	0.11		
**Gender (female vs. male)**	1.56 (0.86–2.66)	0.15			1.30 (0.75–2.26)	0.34		
**Pathological T stage (pT3–4 vs. pT1–2)**	0.65 (0.21–2.01)	0.47			1.63 (0.53–5.00)	1.63		
**Pathological N stage (pN0 vs. pN+)**	0.82 (0.43–1.56)	0.55			0.87 (0.50–1.52)	0.63		
**Surgery (vs. pancreatoduodenectomy)**								
Distal pancreatectomy	0.75 (0.37–1.52)	0.43			0.81 (0.42–1.57)	0.54		
Total pancreatectomy	1.93 (0.59–6.25)	0.28			1.65 (0.52–5.30)	0.40		
**NACT regimens (gemcitabine-based vs. FOLFIRINOX)**	1.22 (0.62–2.40)	0.57			0.99 (0.57–1.72)	0.97		
**CA 19-9 (elevated vs. WNL)**	1.70 (0.92–3.16)	0.09	1.67 (0.96–2.92)	0.07	1.76 (1.00–3.10)	0.05	1.65 (1.00–2.73)	0.049
**Response to NACT (vs. partial response)**								
Stable disease	2.09 (1.03–4.24)	0.04	2.12 (1.16–3.87)	0.02	1.38 (0.78–2.41)	0.27		
Progressive disease	6.59 (1.20–36.33)	0.03	4.92 (1.05–23.04)	0.04	2.59 (0.51–13.06)	0.25		
**Artery resection (yes vs. no)**	1.10 (0.58–2.03)	0.80			1.18 (0.66–2.10)	0.57		
**Vein resection (yes vs. no)**	1.20 (0.60–2.35)	0.63			1.30 (0.69–2.47)	0.42	1.67 (1.01–2.77)	0.047
**Resection margin status (positive vs. negative)**	1.33 (0.66–2.66)	0.43			1.01 (0.53–4.96)	0.98		

HR = hazard ratio, CI = confidence interval, NACT = neoadjuvant chemotherapy, WNL = within normal range.

**Table 5:** Multivariate analyses of overall survival (OS) and disease-free survival (DFS) in the pooled analysis with the inclusion of the conversion surgery after NACT group and upfront surgery group.

Variables	OS from Surgical Resection			DFS from Surgical Resection		
	HR	95% CI	*p*	HR	95% CI	*p*
**Age (≥ 65 vs. <65 years)**	1.17	0.94–1.46	0.15	1.08	0.87–1.34	0.50
**Gender (female vs. male)**	1.07	0.87–1.32	0.51	1.09	0.89–1.33	0.41
**NACT (yes vs. no)**	0.73	0.56–0.96	0.02	0.72	0.56–0.93	0.01
**CA 19-9 (elevated vs. WNL)**	1.22	0.97–1.53	0.09	1.19	0.96–1.49	0.12
**Surgery (vs. pancreatoduodenectomy)**						
Distal pancreatectomy	0.99	0.74–1.33	0.96	1.07	0.81–1.41	0.66
Total pancreatectomy	1.47	1.13–1.91	0.004	1.39	1.07–1.80	0.01

HR = hazard ratio, CI = confidence interval, NACT = neoadjuvant chemotherapy, WNL = within normal range.

## Supplementary Materials

The following are available online at https://www.mdpi.com/2072-6694/11/3/278/s1, Figure S1: Study flow diagram.

## References

[1] Siegel R.L., Miller K.D., Jemal A. (2016). Cancer statistics, 2016. CA Cancer J. Clin..

[2] Jung K.-W., Won Y.-J., Oh C.-M., Kong H.-J., Lee D.H., Lee K.H., The Community of Population-Based Regional Cancer Registries (2017). Cancer Statistics in Korea: Incidence, Mortality, Survival, and Prevalence in 2014. Cancer Res. Treat..

[3] Heestand G.M., Murphy J.D., Lowy A.M. (2015). Approach to Patients with Pancreatic Cancer Without Detectable Metastases. J. Clin. Oncol..

[4] Katz M.H.G., Marsh R., Herman J.M., Shi Q., Collison E., Venook A.P., Kindler H.L., Alberts S.R., Philip P., Lowy A.M. (2013). Borderline Resectable Pancreatic Cancer: Need for Standardization and Methods for Optimal Clinical Trial Design. Ann. Surg. Oncol..

[5] National Comprehensive Cancer Network (2016). Pancreatic Adenocarcinoma (Version 1). Http://www.Nccn.org/Professionals/Physician_Gls/Pdf/Pancreatic.Pdf.

[6] Callery M.P., Chang K.J., Fishman E.K., Talamonti M.S., William T.L., Linehan D.C. (2009). Pretreatment Assessment of Resectable and Borderline Resectable Pancreatic Cancer: Expert Consensus Statement. Ann. Surg. Oncol..

[7] Lee J.-L., Kim S.C., Kim J.-H., Lee S.S., Kim T.-W., Park D.H., Seo D.W., Lee S.K., Kim M.-H., Kim J.H. (2012). Prospective efficacy and safety study of neoadjuvant gemcitabine with capecitabine combination chemotherapy for borderline-resectable or unresectable locally advanced pancreatic adenocarcinoma. Surgery.

[8] O’Reilly E.M., Perelshteyn A., Jarnagin W.R., Schattner M., Gerdes H., Capanu M., Tang L.H., LaValle J., Winston C., DeMatteo R.P. (2014). A Single-Arm, Nonrandomized Phase II Trial of Neoadjuvant Gemcitabine and Oxaliplatin in Patients with Resectable Pancreas Adenocarcinoma. Ann. Surg..

[9] Conroy T., Desseigne F., Ychou M., Bouché O., Guimbaud R., Becouarn Y., Adenis A., Raoul J.-L., Gourgou-Bourgade S., de la Fouchardière C. (2011). FOLFIRINOX versus gemcitabine for metastatic pancreatic cancer. N. Engl. J. Med..

[10] Von Hoff D.D., Ervin T., Arena F.P., Chiorean E.G., Infante J., Moore M., Seay T., Tjulandin S.A., Ma W.W., Saleh M.N. (2013). Increased Survival in Pancreatic Cancer with nab-Paclitaxel plus Gemcitabine. N. Engl. J. Med..

[11] Eisenhauer E.A., Therasse P., Bogaerts J., Schwartz L.H., Sargent D., Ford R., Seay T., Tjulandin S.A., Ma W.W., Saleh M.N. (2009). New response evaluation criteria in solid tumours: Revised RECIST guideline (version 1.1). Eur. J. Cancer.

[12] Dindo D., Demartines N., Clavien P.-A. (2004). Classification of Surgical Complications. Ann. Surg..

[13] Bassi C., Marchegiani G., Dervenis C., Sarr M., Abu Hilal M., Adham M., Allen P., Andersson R., Asbun H.J., Besselink M.G. (2017). The 2016 update of the International Study Group (ISGPS) definition and grading of postoperative pancreatic fistula: 11 Years After. Surgery.

[14] Gemenetzis G., Groot V.P., Blair A.B., Laheru D.A., Zheng L., Narang A.K., Fishman E.K., Hruban R.H., Yu J., Burkhart R.A. (2018). Survival in Locally Advanced Pancreatic Cancer After Neoadjuvant Therapy and Surgical Resection. Ann. Surg..

[15] Neoptolemos J.P., Palmer D.H., Ghaneh P., Psarelli E.E. (2017). Comparison of adjuvant gemcitabine and capecitabine with gemcitabine monotherapy in patients with resected pancreatic cancer (ESPAC-4): A multicentre, open-label, randomised, phase 3 trial. Lancet.

[16] Murphy J.E., Wo J.Y., Ryan D.P., Jiang W., Yeap B.Y., Drapek L.C., Blaszkowsky L.S., Kwak E.L., Allen J.N., Clark J.W. (2018). Total Neoadjuvant Therapy with FOLFIRINOX Followed by Individualized Chemoradiotherapy for Borderline Resectable Pancreatic Adenocarcinoma. JAMA Oncol..

[17] Suker M., Beumer B.R., Sadot E., Marthey L., Faris J.E., Mellon E.A., El-Rayes B.F., Wang-Gillam A., Lacy J., Hosein P.J. (2016). FOLFIRINOX for locally advanced pancreatic cancer: a systematic review and patient-level meta-analysis. Lancet Oncol..

[18] Khushman M., Dempsey N., Maldonado J.C., Loaiza-Bonilla A., Velez M., Carcas L., Dammrich D., Hurtado-Cordovi J., Parajuli R., Pollack T. (2015). Full dose neoadjuvant FOLFIRINOX is associated with prolonged survival in patients with locally advanced pancreatic adenocarcinoma. Pancreatology.

[19] Yoo C., Kang J., Kim K.-P., Lee J.-L., Ryoo B.-Y., Chang H.-M., Lee S.S., Park D.H., Song T.J., Seo D.W. (2017). Efficacy and safety of neoadjuvant FOLFIRINOX for borderline resectable pancreatic adenocarcinoma: Improved efficacy compared with gemcitabine-based regimen. Oncotarget.

[20] Katz M.H.G., Ou F.-S., Herman J.M., Ahmad S.A., Wolpin B., Marsh R., Behr S., Shi Q., Chuong M., Schwartz L.H. (2017). Alliance for clinical trials in oncology (ALLIANCE) trial A021501: Preoperative extended chemotherapy vs. chemotherapy plus hypofractionated radiation therapy for borderline resectable adenocarcinoma of the head of the pancreas. BMC Cancer.

[21] Ferrone C.R., Marchegiani G., Hong T.S., Ryan D.P., Deshpande V., McDonnell E.I., Sabbatino F., Santos D.D., Allen J.N., Blaszkowsky L.S. (2015). Radiological and Surgical Implications of Neoadjuvant Treatment with FOLFIRINOX for Locally Advanced and Borderline Resectable Pancreatic Cancer. Ann. Surg..

[22] Mokdad A.A., Minter R.M., Zhu H. (2016). Neoadjuvant therapy followed by resection versus upfront resection for resectable pancreatic cancer: A propensity score matched analysis. J. Clin. Oncol..

[23] Conroy T., Hammel P., Hebbar M., Ben Abdelghani M., Wei A.C.-C., Raoul J.-L., Chone L., Francois E., Artru P., Biagi J.J. (2018). Unicancer GI PRODIGE 24/CCTG PA.6 trial: A multicenter international randomized phase III trial of adjuvant mFOLFIRINOX versus gemcitabine (gem) in patients with resected pancreatic ductal adenocarcinomas. J. Clin. Oncol..

