# Supplementary material for: Clinical Outcomes of Conversion Surgery after Neoadjuvant Chemotherapy in Patients with Borderline Resectable and Locally Advanced Unresectable Pancreatic Cancer: A Single-Center, Retrospective Analysis
Source: Cancers (Basel). 2019 Feb 26;11(3):278. doi: 10.3390/cancers11030278 (PMC6468804; doi:10.3390/cancers11030278)
Supplement: Supplementary file 1 [file cancers-11-00278-s001.pdf]

cancers

MDPI

Supplementary Materials

Clinical Outcomes of Conversion Surgery after Neoadjuvant Chemotherapy in Patients with Borderline Resectable and Locally Advanced Unresectable Pancreatic Cancer: A Single-Center, Retrospective Analysis

Changhoon Yoo, Sang Hyun Shin, Kyu-pyo Kim, Jae Ho Jeong, Heung-Moon Chang, Jun Ho Kang, Sang Soo Lee, Do Hyun Park, Tae Jun Song, Dong Wan Seo, Sung Koo Lee, Myung-Hwan Kim, Jin-hong Park, Dae Wook Hwang, Ki Byung Song, Jae Hoon Lee, Baek-Yeol Ryoo and Song Cheol Kim

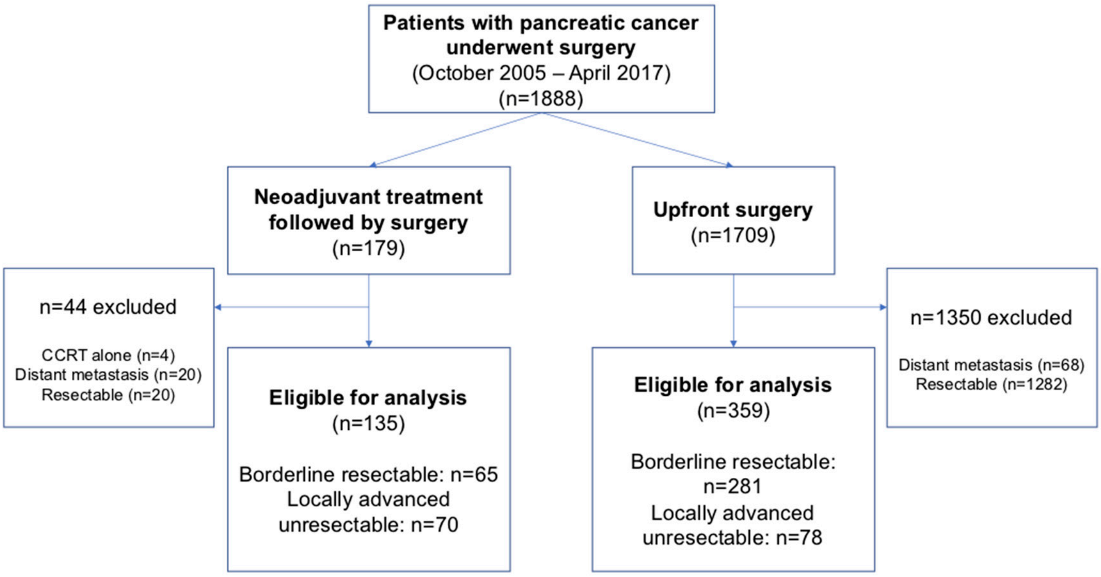

Figure S1. Study flow diagram.
